# Risks of Placental Abruption and Preterm Delivery in Patients Undergoing Assisted Reproduction

**DOI:** 10.1001/jamanetworkopen.2024.20970

**Published:** 2024-07-10

**Authors:** Jennifer T. Zhang, Rachel Lee, Mark V. Sauer, Cande V. Ananth

**Affiliations:** 1 Department of Obstetrics, Gynecology, and Reproductive Sciences, Rutgers Robert Wood Johnson Medical School, New Brunswick, New Jersey; 2 Division of Epidemiology and Biostatistics, Department of Obstetrics, Gynecology, and Reproductive Sciences, Rutgers Robert Wood Johnson Medical School, New Brunswick, New Jersey; 3 Department of Biostatistics and Epidemiology, Rutgers School of Public Health, Piscataway, New Jersey; 4 Department of Medicine, Rutgers Robert Wood Johnson Medical School, New Brunswick, New Jersey; 5 Cardiovascular Institute, Rutgers Robert Wood Johnson Medical School, New Brunswick, New Jersey

## Abstract

**Importance:**

Patients using assisted reproductive technology (ART) may need additional counseling about the increased risks of placental abruption and preterm delivery. Further investigation into the potential additive risk of ART and placental abruption is needed.

**Objective:**

To ascertain the risk of placental abruption in patients who conceived with ART and to evaluate if placental abruption and ART conception are associated with an increased risk of preterm delivery (<37 weeks’ gestation) over and above the risks conferred by each factor alone.

**Design, Setting, and Participants:**

This cross-sectional study used data from the National Inpatient Sample, which includes data from all-payer hospital inpatient discharges from 48 states across the US. Participants included women aged 15 to 54 years who delivered from 2000 through 2019. Data were analyzed from January 17 to April 18, 2024.

**Exposures:**

Pregnancies conceived with ART.

**Main Outcomes and Measures:**

Risks of placental abruption and preterm delivery in ART conception compared with spontaneous conceptions. Associations were expressed as odds ratios (ORs) and 95% CIs derived from weighted logistic regression models before and after adjusting for confounders. The relative excess risk due to interaction (RERI) of the risk of preterm delivery based on ART conception and placental abruption was also assessed.

**Results:**

Of 78 901 058 deliveries, the mean (SD) maternal age was 27.9 (6.0) years, and 9 212 117 patients (11.7%) were Black individuals, 14 878 539 (18.9%) were Hispanic individuals, 34 899 594 (44.2%) were White individuals, and 19 910 807 (25.2%) were individuals of other races and ethnicities. Of the total hospital deliveries, 98.2% were singleton pregnancies, 68.8% were vaginal deliveries, and 52.1% were covered by private insurance. The risks of placental abruption among spontaneous and ART conceptions were 11 and 17 per 1000 hospital discharges, respectively. After adjusting for confounders, the adjusted OR (AOR) of placental abruption was 1.42 (95% CI, 1.34-1.51) in ART pregnancies compared with spontaneous conceptions, with increased odds in White women (AOR, 1.42; 95% CI, 1.31-1.53) compared with Black women (AOR, 1.16; 95% CI, 0.93-1.44). The odds of preterm delivery were significantly higher in pregnancies conceived by ART compared with spontaneous conceptions (AOR, 1.46; 95% CI, 1.42-1.51). The risk of preterm delivery increased when patients had both ART conception and placental abruption (RERI, 2.0; 95% CI, 0.5-3.5).

**Conclusions and Relevance:**

In this cross-sectional study, patients who conceived using ART and developed placental abruption had a greater risk of preterm delivery compared with spontaneous conception without placental abruption. These findings have implications for counseling patients who seek infertility treatment and obstetrical management of ART pregnancies.

## Key Points

**Question:**

Is the combination of conception by assisted reproductive technology (ART) and placental abruption associated with the risk of preterm delivery (<37 weeks’ gestation)?

**Findings:**

In this cross-sectional study of 78 901 058 US women who delivered between 2000 and 2019, ART conception had an increased risk of placental abruption and preterm delivery compared with spontaneous conception. There was an additive risk of preterm delivery in ART conception with placental abruption compared with ART or abruption alone.

**Meaning:**

These findings suggest that patients who conceive using ART and then develop placental abruption have a greater risk of preterm delivery, which has consequences for the neonate.

## Teaser

This cross-sectional study examines the risks of preterm delivery associated with assisted reproduction and placental abruption alone or in combination.

## Introduction

Worldwide, assisted reproductive technology (ART) is being increasingly used for infertility treatment. The Centers for Disease Control and Prevention reported that 238 126 patients underwent 413 776 ART cycles in the US in 2021, resulting in 91 906 live births.^1^ As delayed childbearing and improved access to ART become more common, these numbers will likely increase. Assisted reproductive technology includes in vitro fertilization, cryopreservation of gametes or zygotes, intracytoplasmic sperm injection (ICSI), frozen embryo transfer (FET) cycles, and preimplantation genetic testing. However, pregnancies resulting from ART are associated with neonatal complications, including preterm delivery, small for gestational age (SGA) births, and neonatal and infant mortality.^2,3,4^ Antenatally, these pregnancies carry increased risks of placental abruption, placenta previa, preeclampsia, gestational diabetes, cesarean delivery, and short-term risks of heart disease and stroke.^5,6,7,8,9,10^

Placental abruption is the premature detachment of the placenta from the uterine wall before delivery.^11,12^ In one study, singleton pregnancies conceived with ART experienced an 83% increase in abruption compared with spontaneously conceived pregnancies.^13^ In another study, patients who underwent ovulation induction or used ART were 2.4 times more likely to have abruption.^14^ Women with abruption have an increased risk of preterm labor compared with those with premature rupture of membranes or a medical indication for preterm delivery.^15^ Perinatal mortality risks among births with or without placental abruption were 102.7 and 6.2 per 1000 births, respectively, which further increased at earlier gestational ages.^16^ Given how ART, placental abruption, and preterm delivery affect neonatal morbidity and mortality, there is limited literature on the association among these 3 entities.

The use of ART can introduce processes that are not characteristic of in vivo reproduction. For example, ART may increase the risk of placental abnormalities due to oxidative, thermal, and mechanical stresses on molecules already undergoing epigenetic remodeling.^17^ While the exact pathophysiologic mechanisms are unknown, these conditions may arise from a similar etiology of inadequate remodeling of spiral arteries in early pregnancy causing uteroplacental ischemia.^18,19^ Studies have shown the rate of preterm delivery increases as the pregnancy progresses, often due to medical indications—most commonly placental abruption, preeclampsia, and SGA birth, collectively referred to as ischemic placental disease (IPD).^20^ In one study,^21^ ART pregnancies had 4 times the risk of preterm IPD compared with pregnancies not involving ART.

Racial disparities in the development of placental abruption and access to ART contribute to inequities in obstetric and neonatal outcomes.^22,23,24,25^ In the US, chronic hypertension affects a greater percentage of reproductive-age Black women (15%-19%) than White women (6%-10%) or Hispanic women (3%-10%),^25^ which contributes to higher rates of placental abruption, preterm delivery, superimposed preeclampsia, cerebrovascular accidents, pulmonary edema, acute kidney failure, and pregnancy-related death. Hispanic women also have higher rates of placental abruption, eclampsia, preterm birth, and neonatal death compared with non-Hispanic White women despite the former having a lower risk of chronic hypertension.^22^ These disparities may be explained by similar differences in social determinants of health and access to care and insurance coverage.^22^ Fewer Black women are undergoing ART treatment despite an increasing prevalence of infertility among Black women and a decreasing prevalence of infertility among White women.^26^ We designed this large population-based study to determine the risk of placental abruption in relation to infertility treatment and to evaluate if exposures to both placental abruption and ART are associated with increased risk of preterm delivery (<37 weeks’ gestation) beyond the risks conferred by each factor independently and stratified by race and ethnicity.

## Methods

Data were obtained from the National Inpatient Sample (NIS) as part of the Healthcare Cost and Utilization Project (HCUP), sponsored by the Agency for Healthcare Research and Quality. The NIS is the largest US database of inpatient hospital stays from all HCUP-participating hospitals (28 states and 994 hospitals in 2000), with more states and hospitals participating in HCUP over time (48 states plus the District of Columbia and 4568 hospitals in 2019). The NIS encompasses 98% of the US population and accounts for 7 to 8 million unweighted hospital discharges and 36 to 39 million weighted hospital discharges each year. This cross-sectional study followed the Strengthening the Reporting of Observational Studies in Epidemiology (STROBE) reporting guideline^27^ and, in accordance with 45 CFR §46, was deemed exempt from institutional review board review and the informed consent requirement because data used in the study were deidentified.

Statistically weighted discharge data, estimated within each stratum defined by hospital characteristics—census division, rural vs urban location, hospital bed size, teaching status, and hospital ownership—were provided by the NIS for calculating national estimates. The NIS reports data with diagnosis and procedure codes from the *International Classification of Diseases, Ninth Revision, Clinical Modification *(*ICD-9-CM*) (from 2000 through third quarter 2015) and *International Statistical Classification of Diseases, Tenth Revision, Clinical Modification *(*ICD-10-CM*) (from fourth quarter 2015 through 2019) coding system, which enabled us to determine hospitalized deliveries, ART procedures, placental abruption, and preterm delivery (eTable 1 in Supplement 1). The cohort consisted of women aged 15 to 54 years who delivered in the hospital from 2000 through 2019 regardless of delivery outcome (live birth or stillbirth) and plurality (singleton or multiple births).

The HCUP provided a uniform code for race and ethnicity based on original data from HCUP partner organizations. In the NIS, race and ethnicity were coded as Asian or Pacific Islander, Black, Hispanic, Native American, White, or other (not defined in the NIS dataset), and ethnicity took precedence over race. For example, a patient who was Hispanic and Black was assigned to the category of Hispanic.^28^ We evaluated 4 categories of race and ethnicity: Black, Hispanic, White, and other (including Asian or Pacific Islander, Native American, and other race and ethnicity).

### Covariates

We adjusted for potential confounding by maternal age at delivery (15-19, 20-24, 25-29, 30-34, 35-39, 40-44, and 45-54 years), hospital bed size (small, medium, or large) specific to the hospital’s location and teaching status,^29^ hospital teaching status (teaching, nonteaching, or status unknown), hospital location (rural, urban, or unknown), region (Northeast, Midwest, South, or West), race and ethnicity (Black, Hispanic, White, or other), plurality (singleton or multiple births), income quartile (low, medium-low, medium-high, high, or unknown; derived from median household income by zip code and varies over time), insurance (Medicare, Medicaid, private, self-pay, other, or unknown),^30^ and year of delivery. Sample weights, clustering, and stratification were used to provide nationally representative estimates.

### Statistical Analysis

We estimated the overall risks of placental abruption and preterm delivery by race, plurality, age group, and income quartile among patients who conceived with ART compared with those who conceived spontaneously, as well as the risks of preterm delivery among patients with spontaneous conceptions, ART conception, and placental abruption. We fit a logistic regression model to estimate the unadjusted and confounder-adjusted odds ratios (ORs and AORs, respectively) and 95% CIs stratified by the mother’s race and ethnicity.

To estimate the biological interactions of ART conception and placental abruption with preterm delivery, we calculated the relative risk excess due to interaction (RERI) among patients with ART conception and placental abruption. The RERI describes departure from outcomes on a relative risk (RR) scale. A positive RERI implies that the number of cases attributable to 2 hazards in combination is larger than the sum of the numbers of cases that would be caused by each hazard separately.^31^ The formula to calculate the RERI is: RERI = RR_11_ – RR_10_ – RR_01_ + 1, where RR_ab_ is the RR in the group with X_1_ exposure status a (1 = exposed; 0 = unexposed), and X_2_ exposure status b (1 = exposed; 0 = unexposed) compared with the double-exposed group.^32^ An RERI of 0 implies no additive interaction, RERI greater than 0 implies a superadditive interaction, and RERI less than 0 implies a subadditive interaction.^33^

The statistical analysis was performed in SAS, version 9.4 (SAS Institute Inc). Data were analyzed from January 17 to April 18, 2024.

## Results

Of the 78 901 058 hospital deliveries between 2000 and 2019, 391 780 (0.5%) of pregnancies were conceived with ART (Table 1). The mean (SD) maternal age was 27.9 (6.0) years, and 9 212 117 patients (11.7%) were Black individuals, 14 878 539 (18.9%) were Hispanic individuals, 34 899 594 (44.2%) were White individuals, and 19 910 807 (25.2%) were individuals of other races and ethnicities. Of the total hospital deliveries, 98.2% were singleton pregnancies, 68.8% were vaginal deliveries, and 52.1% were covered by private insurance. The prevalence of pregnancies conceived with ART increased from 2000 to 2019, and the rate of placental abruption in ART conceptions was about 2-fold higher than in spontaneous conceptions but declined over time (eFigure in Supplement 1).

**Table 1.** Distribution of Women Who Conceived by ART and Those Who Conceived Spontaneously From the National Inpatient Sample, 2000 to 2019

Characteristics	Women, No. (%)		
	Total delivery hospitalizations	ART conception	Spontaneous conception
All patients	78 901 058 (100)	391 780 (100)	78 509 278 (100)
Age at delivery, y			
15-19	6 751 463 (8.6)	2013 (0.5)	6 749 449 (8.6)
20-24	18 281 716 (23.2)	22 910 (5.8)	18 258 807 (23.3)
25-29	22 000 268 (27.9)	84 224 (21.5)	21 916 044 (27.9)
30-34	19 838 243 (25.1)	141 113 (36.0)	19 697 130 (25.1)
35-39	9 796 486 (12.4)	98 579 (25.1)	9 697 907 (12.4)
40-44	2 101 349 (2.6)	34 297 (8.8)	2 067 052 (2.6)
45-54	131 534 (0.2)	8644 (2.2)	122 891 (0.2)
Maternal race and ethnicity			
Black	9 212 117 (11.7)	25 471 (6.5)	9 186 646 (11.7)
Hispanic	14 878 539 (18.9)	33 318 (8.5)	14 845 220 (18.9)
White	34 899 594 (44.2)	248 204 (63.4)	34 651 391 (44.1)
Other^a^	19 910 807 (25.2)	84 787 (21.6)	19 826 021 (25.3)
Plurality			
Singleton	77 490 952 (98.2)	336 512 (85.9)	77 154 440 (98.3)
Multiple	1 410 106 (1.8)	55 268 (14.1)	1 354 838 (1.7)
Mode of delivery			
Vaginal	54 321 703 (68.8)	189 342 (48.3)	54 132 361 (69.0)
Cesarean	24 579 354 (31.2)	202 438 (51.7)	24 376 917 (31.0)
Hospital teaching status			
Teaching	37 630 315 (47.7)	115 470 (29.5)	37 514 845 (47.8)
Nonteaching	41 032 773 (52.0)	275 600 (70.3)	40 757 174 (51.9)
Unknown	237 970 (0.3)	711 (0.2)	237 259 (0.3)
Hospital location			
Rural	8 785 417 (11.1)	22 621 (5.8)	8 762 796 (11.2)
Urban	69 877 671 (88.6)	368 449 (94.0)	69 509 223 (88.5)
Unknown	237 970 (0.3)	711 (0.2)	237 259 (0.3)
Hospital bed size^b^			
Small	10 334 292 (13.1)	56 286 (14.4)	10 278 006 (13.1)
Medium	21 872 192 (27.7)	103 889 (26.5)	21 768 303 (27.7)
Large	46 456 604 (58.9)	230 894 (58.9)	46 225 710 (58.9)
Unknown	237 970 (0.3)	711 (0.2)	237 259 (0.3)
Region			
Northeast	12 910 828 (16.4)	100 286 (25.6)	12 810 542 (16.3)
Midwest	16 880 053 (21.4)	90 098 (23.0)	16 789 955 (21.4)
South	29 970 866 (38.0)	108 658 (27.7)	29 862 209 (38.0)
West	19 139 310 (24.3)	92 739 (23.7)	19 046 571 (24.3)
Family income			
Low	18 863 149 (23.9)	51 207 (13.1)	18 811 942 (24.0)
Medium-low	19 098 386 (24.2)	73 684 (18.8)	19 024 702 (24.2)
Medium-high	19 226 420 (24.4)	103 790 (26.5)	19 122 630 (24.4)
High	20 491 818 (26.0)	159 723 (40.8)	20 332 096 (25.9)
Unknown	1 221 285 (1.5)	3376 (0.9)	1 217 909 (1.6)
Insurance			
Medicare	464 324 (0.6)	2072 (0.5)	462 252 (0.6)
Medicaid	32 562 238 (41.4)	47 579 (12.1)	32 254 659 (41.4)
Private	41 122 695 (52.1)	327 222 (83.5)	40 795 474 (52.0)
Self-pay	2 406 605 (3.1)	4591 (1.2)	2 402 014 (3.1)
Other^c^	2 213 839 (2.8)	9828 (2.5)	2 204 011 (2.8)
Unknown	131 356 (0.2)	488 (0.1)	130 868 (0.2)
Year of delivery			
2000	4 044 942 (5.1)	3512 (0.9)	4 041 430 (5.1)
2001	3 948 636 (5.0)	4118 (1.1)	3 944 518 (5.0)
2002	4 105 431 (5.2)	6532 (1.7)	4 098 899 (5.2)
2003	4 028 568 (5.1)	5557 (1.4)	4 023 011 (5.1)
2004	4 186 922 (5.3)	8259 (2.1)	4 178 663 (5.3)
2005	4 174 744 (5.5)	9804 (2.5)	4 164 940 (5.3)
2006	4 243 821 (5.4)	7380 (1.9)	4 236 442 (5.4)
2007	4 501 441 (5.7)	9694 (2.5)	4 491 747 (5.7)
2008	4 193 566 (5.3)	13 204 (3.4)	4 180 362 (5.3)
2009	4 102 423 (5.2)	16 139 (4.1)	4 086 283 (5.2)
2010	3 850 536 (4.9)	18 756 (4.8)	3 831 781 (4.9)
2011	3 803 473 (4.8)	26 119 (6.7)	3 777 354 (4.8)
2012	3 759 856 (4.8)	24 060 (6.1)	3 735 796 (4.8)
2013	3 736 073 (4.7)	27 745 (7.1)	3 708 328 (4.7)
2014	3 796 470 (4.8)	33 545 (8.6)	3 762 925 (4.8)
2015	3 799 425 (4.8)	39 200 (10.0)	3 760 225 (4.8)
2016	3 756 481 (4.7)	45 240 (11.5)	3 711 241 (4.7)
2017	3 679 726 (4.6)	30 000 (7.7)	3 649 726 (4.6)
2018	3 614 469 (4.6)	29 730 (7.6)	3 584 739 (4.6)
2019	3 574 054 (4.5)	33 185 (8.5)	3 540 869 (4.5)

Abbreviation: ART, assisted reproductive technology.

^a^ Other includes Asian or Pacific Islander, Native American, and other (not defined in National Inpatient Sample dataset).

^b^ Definitions of hospital bed size vary by region, rural vs urban status, and technical status.^29^

^c^ Definition of other varies by state.^30^

The risks of placental abruption among spontaneous and ART conceptions are listed in Table 2. The overall risk of placental abruption in pregnancies conceived by ART was higher than that in spontaneously conceived pregnancies (17 vs 11 per 1000 births, respectively). After adjusting for confounders, the risk of placental abruption was higher in all ART pregnancies compared with spontaneous pregnancies (AOR, 1.42; 95% CI, 1.34-1.51).

**Table 2.** Risks of Placental Abruption by Race and Ethnicity Among Patients Stratified by ART or Spontaneous Conception, National Inpatient Sample, 2000 to 2019

Race and ethnicity	ART conception		Spontaneous conception		Risk difference (95% CI) per 1000 delivery hospitalizations	Odds ratio (95% CI)	
	Total delivery hospitalizations, No.	Placental abruption (risk per 1000 delivery hospitalizations)	Total delivery hospitalizations, No.	Placental abruption (risk per 1000 delivery hospitalizations)		Unadjusted	Adjusted
Overall^a^	391 780	6588 (17)	78 509 278	844 092 (11)	6 (5 to 7)	1.57 (1.48 to 1.68)	1.42 (1.34 to 1.51)
Black^b^	25 471	456 (18)	9 186 646	135 082 (15)	3 (−1 to 1)	1.22 (0.98 to 1.52)	1.16 (0.93 to 1.44)
Hispanic^b^	33 318	633 (19)	14 845 220	139 716 (9)	10 (6 to 13)	2.04 (1.70 to 2.45)	1.66 (1.38 to 1.99)
White^b^	248 204	3937 (16)	34 651 391	352 309 (10)	6 (4 to 7)	1.57 (1.48 to 1.70)	1.42 (1.31 to 1.53)
Other^b^^,^^c^	84 787	1563 (18)	19 826 021	216 985 (11)	8 (5 to 10)	1.70 (1.51 to 1.91)	1.49 (1.33 to 1.68)

Abbreviation: ART, assisted reproductive technology.

^a^ Overall odds ratios were adjusted for year, age group, hospital bed size, hospital teaching status, location, region, maternal race and ethnicity, singleton vs multiple births, insurance, and income based on a survey logistic regression model.

^b^ Odds ratios by maternal race and ethnicity were adjusted for year, age group, hospital bed size, hospital teaching status, location, region, singleton vs multiple birth, insurance, and income based on a survey logistic regression model.

^c^ Other includes Asian or Pacific Islander, Native American, and other (not defined in National Inpatient Sample dataset).

The risks of preterm delivery among spontaneous and ART conceptions, stratified by race, are listed in Table 3. Overall, the risk of preterm delivery was greater for pregnancies conceived by ART compared with spontaneous conception (AOR, 1.46; 95% CI, 1.42-1.51).

**Table 3.** Risks of Preterm Delivery Associated With ART vs Spontaneous Conception and Stratified by Race and Ethnicity, National Inpatient Sample, 2000 to 2019^a^

Race and ethnicity	ART conception		Spontaneous conception		Risk difference (95% CI) per 1000 delivery hospitalizations	Odds ratio (95% CI)	
	Total delivery hospitalizations, No.	Preterm delivery (risk per 1000 delivery hospitalizations)	Total delivery hospitalizations, No.	Preterm delivery (risk per 1000 delivery hospitalizations)		Unadjusted	Adjusted^b^
Overall	391 780	63 724 (163)	78 509 278	6 043 032 (77)	86 (81-90)	2.33 (2.25-2.41)	1.46 (1.42-1.51)
Black	25 471	5330 (209)	9 186 646	1 026 809 (112)	98 (86-109)	2.10 (1.95-2.26)	1.51 (1.39-1.64)
Hispanic	33 318	5960 (179)	14 845 220	1 103 546 (74)	105 (95-115)	2.72 (2.54-2.91)	1.62 (1.51-1.75)
White	248 204	38 695 (156)	34 651 391	2 455 695 (71)	85 (79-91)	2.42 (2.32-2.53)	1.46 (1.41-1.52)
Other^c^	84 787	13 738 (162)	19 826 021	1 456 983 (73)	89 (81-96)	2.44 (2.30-2.58)	1.42 (1.35-1.49)

Abbreviation: ART, assisted reproductive technology.

^a^ Preterm delivery occurred before 37 weeks’ gestation.

^b^ Odds ratios were adjusted for year, age group, hospital bed size, hospital teaching status, location, region, singleton vs multiple births, insurance, and income based on a survey logistic regression model (and additionally adjusted for mother’s race and ethnicity for overall associations).

^c^ Other includes Asian or Pacific Islander, Native American, and other (not defined in National Inpatient Sample dataset).

The risk of preterm delivery was greater in patients with both ART conception and placental abruption than in patients who experienced only 1 factor (555 per 1000 births vs 156 per 1000 births for ART conception alone and 421 per 1000 births for placental abruption alone) (Table 4). The RERI analysis showed an increase in preterm deliveries when patients had both ART conception and placental abruption (RERI, 2.0; 95% CI, 0.5-3.5) (Table 5). This association was attenuated in Black and Hispanic women.

**Table 4.** Preterm Delivery Risks Stratified by Race and Ethnicity Based on Conception by ART Alone, Placental Abruption Alone, or Both Factors, National Inpatient Sample, 2000 to 2019

Race and ethnicity	ART conception only		Spontaneous conception		Placental abruption only		Both ART conception and placental abruption	
	Total delivery hospitalizations, No.	Preterm delivery (risk per 1000 delivery hospitalizations)	Total delivery hospitalizations, No.	Preterm delivery (risk per 1000 delivery hospitalizations)	Total pregnancies, No.	Preterm delivery (risk per 1000 delivery hospitalizations)	Total delivery hospitalizations, No.	Preterm delivery (risk per 1000 delivery hospitalizations)
Overall	385 192	60 065 (156)	77 665 186	5 687 626 (73)	844 092	355 406 (421)	6588	3659 (555)
Black	25 015	5078 (203)	9 051 564	961 112 (106)	135 082	65 688 (486)	456	253 (554)
Hispanic	32 686	5588 (171)	14 705 504	1 044 310 (71)	139 716	59 236 (424)	633	373 (589)
White	244 267	36 506 (149)	34 299 081	2 313 037 (67)	352 309	142 658 (405)	3937	2189 (556)
Other^a^	83 224	12 894 (155)	19 609 036	1 369 158 (70)	216 985	87 825 (405)	1563	845 (541)

Abbreviation: ART, assisted reproductive technology.

^a^ Other includes Asian or Pacific Islander, Native American, and other (not defined in National Inpatient Sample dataset).

**Table 5.** Association of Conception by ART and Placental Abruption, Alone and in Combination, With the Risk of Preterm Delivery by Race and Ethnicity, National Inpatient Sample, 2000 to 2019^a^

Race and ethnicity	Odds ratio (95% CI)						RERI (95% CI)
	ART conception only		Placental abruption only		Both ART conception and placental abruption		
	Unadjusted	Adjusted^a^	Unadjusted	Adjusted^a^	Unadjusted	Adjusted^a^	
Overall	2.34 (2.26 to 2.42)	1.45 (1.41 to 1.50)	9.20 (9.06 to 9.35)	9.27 (9.12 to 9.42)	15.81 (14.09 to 17.73)	11.76 (10.34 to 13.38)	2.0 (0.5 to 3.5)
Black	2.14 (1.99 to 2.31)	1.52 (1.40 to 1.65)	7.97 (7.74 to 8.20)	8.14 (7.90 to 8.39)	10.47 (7.00 to 15.64)	9.10 (5.97 to 13.88)	0.4 (−3.4 to 4.3)
Hispanic	2.70 (2.52 to 2.89)	1.61 (1.50 to 1.74)	9.63 (9.34 to 9.93)	9.86 (9.55 to 10.18)	18.70 (13.03 to 26.82)	11.25 (7.63 to 16.60)	0.8 (−3.6 to 5.1)
White	2.43 (2.33 to 2.53)	1.45 (1.40 to 1.51)	9.41 (9.22 to 9.60)	9.56 (9.37 to 9.76)	17.31 (14.95 to 20.03)	12.63 (10.67 to 14.96)	2.6 (0.5 to 4.7)
Other^b^	2.44 (2.30 to 2.59)	1.40 (1.33 to 1.48)	9.06 (8.77 to 9.36)	9.09 (8.81 to 9.37)	15.70 (12.53 to 19.62)	11.25 (8.59 to 14.75)	1.8 (−1.3 to 4.8)

Abbreviations: ART, assisted reproductive technology; RERI, relative excess risk due to interaction.

^a^ Odds ratios were adjusted for year, age group, hospital bed size, hospital teaching status, location, region, singleton vs multiple births, insurance, and income based on a survey logistic regression model (and additionally adjusted for mother’s race and ethnicity for overall associations).

^b^ Other includes Asian or Pacific Islander, Native American, and other (not defined in National Inpatient Sample dataset).

Results according to plurality, age group, and income are included in eTables 2 through 5 in Supplement 1. The risks of placental abruption in pregnancies conceived by ART were higher for singleton pregnancies (AOR, 1.46; 95% CI, 1.37-1.56), women aged 25 to 29 years (AOR, 1.63; 95% CI, 1.45-1.84), and in women with high incomes (AOR, 1.62; 95% CI, 1.48-1.78). Risks of preterm delivery in ART conceptions were higher in singleton pregnancies (AOR 1.62; 95% CI, 1.57-1.67), patients aged 20 to 24 years (AOR, 1.65; 95% CI, 1.51-1.79), and in women with medium to high incomes (AOR, 1.49; 95% CI, 1.42-1.56). Preterm delivery rates in pregnancies with both ART conception and placental abruption were highest in multiple-gestation pregnancies (769 per 1000 births), women aged 45 to 54 years (618 per 1000 births), and women with low to medium incomes (590 per 1000 births). The RERI for preterm delivery in pregnancies with ART conception and placental abruption was highest in singleton pregnancies (RERI, 3.8; 95% CI, 2.0-5.5), women aged 30 to 34 years (RERI, 3.7; 95% CI, 0.9-6.5), and women with low to medium incomes (RERI, 5.1; 95% CI, 0.5-9.6).

## Discussion

In this large epidemiological cross-sectional study, women who conceived by ART had an increased risk of placental abruption, and women who had both ART conception and placental abruption had an increased risk of preterm delivery compared with women with only 1 of these 2 risk factors. Our study is unique, to our knowledge, in that we explored the joint contributions of ART conception and placental abruption on the risk of preterm delivery.

### ART and Placental Abruption

Among preterm births, ART conception has been associated with higher rates of placental infarction after adjusting for confounding factors, such as hypertensive disorders and fetal growth restriction.^34^ This brings into question: are placental abnormalities due to the processes of ART itself or due to the underlying infertility? A meta-analysis^35^ evaluating abnormal placentation between ART conception vs spontaneous conception in patients with reduced fertility compared with a randomized population showed a smaller effect size, suggesting that decreased fertility likely contributes to placental disease. Patients with infertility may have hormonal dysfunction including hyperandrogenism, progesterone resistance, and hyperinsulinism, which appear to impair uterine placental mechanisms.^36^

Other studies have found that placentas in ART conceptions had increased placental thickness, suggesting placental pathology is due to ART rather than patient factors.^37^ Women who become pregnant via ART are exposed to supraphysiologic estradiol levels during ART cycles. Estradiol downregulates GATA3, a transcription factor expressed in trophectoderm during embryonic development, and decreased GATA3 leads to impaired trophoblast cell migration through downstream genes integral in placentation.^38^ Additionally, abnormal *H19* methylation imprints were found in ART-derived human preimplantation embryos.^39^ In a mouse model, loss of *H19* imprinting affected placental tissues more than embryonic tissue.^40^ In humans, levels of pregnancy-associated plasma protein A, a protein involved in trophoblast invasion by contributing to maternal tolerance toward the fetus, were decreased in first-trimester pregnancies conceived by ART.^41^ Altogether, evidence suggests an association between abnormal hormonal levels influencing epigenetics and impaired trophoblastic invasion via ART processes.

### ART and Preterm Delivery

While it has been widely reported that ART is associated with an increase in the risk of preterm delivery,^42^ there is no consensus on what specific ART processes confer the greatest risk. In one study, the risk of preterm delivery was 1.5 times greater after ART conception compared with spontaneous conception (11.5% vs 7.7%), but there was no difference in preterm delivery rates with or without ICSI.^43^ In contrast, another study found an increased risk of medically indicated preterm delivery at 32 to 36 weeks’ gestation in ART pregnancies without ICSI compared with ART pregnancies in which ICSI was used (OR, 1.6; 95% CI, 1.1-2.2).^44^ The transfer of embryos at the blastocyst stage (day 5 or 6) is now preferred over transfer at the cleavage stage (day 3) due to increased pregnancy and decreased rates of multifetal gestation.^45^ Studies also suggest that blastocyst transfers and frozen-thawed transfers increase the risk of preterm delivery by 30% to 40%,^46^ likely due to greater in vitro manipulation.

Frozen embryo transfer has been associated with decreased rates of IPD compared with fresh embryo transfers (RR, 0.75; 95% CI, 0.59-0.97),^47^ which was attributable to lower rates of SGA infants (RR, 0.36; 95% CI, 0.16-0.79).^47^ A meta-analysis^48^ reported an increased risk of placental abruption in natural and programmed FET cycles (OR, 1.23; 95% CI, 0.81-1.89). However, there was an increase in IPD in stimulation cycles compared with natural cycles (OR, 1.16; 95% CI, 1.11-2.07) and an increase in preeclampsia in cycles using hormone replacement therapy vs natural cycles (OR, 4.41; 95% CI, 1.25-15.51).^49^ Each cycle received a similar control for ovarian hyperstimulation, so these findings were likely due to hormonal fluctuations, particularly estrogen. Ovarian hyperstimulation in ART was associated with an increased rate of placental abruption (OR, 6.31; 95% CI, 1.44-27.56).^50^ Again, controlled ovarian hyperstimulation is achieved with supraphysiologic levels of estrogen. Superovulation has been associated with altered gene expression in endometrial remodeling during early implantation.^51^

It is uncertain if the increased risks of preterm delivery associated with ART conception and placental abruption are due to ART itself or to complications resulting from ART. These technologies may directly influence placental abnormalities, epigenetic and genetic changes due to environmental manipulation, and underlying subfertility.^52^ One study found that manipulation of mouse embryos in vitro affected blastocyst gene expression, potential for implantation, and placental development.^41^ On the other hand, ART pregnancies have been associated with an adverse maternal-fetal environment,^53^ likely from compensatory mechanisms, which seem to have fewer implications for preterm delivery compared with fertility treatment or preterm delivery mediated by multiple gestations after ART. As single FET cycles are increasingly used that less frequently produce multiple gestations, the roles of direct factors from ART and compensatory factors from an adverse maternal-fetal environment may play a larger role. These unknown factors highlight the need for ongoing research on the mechanisms of preterm delivery, therapeutic targets, and modulating clinical practice.

We found a smaller increased risk of placental abruption in Black patients undergoing ART compared with the risk among White and Hispanic patients. The RERI analysis indicated that the risks of preterm delivery for the combination of ART conception and placental abruption were higher in preterm delivery among Black and Hispanic patients, but substantially higher among White patients. Previous epidemiological studies found that Black race was a risk factor for placental abruption.^54,55,56^ Preterm delivery rates may differ among races due to psychosocial stressors, epigenetic factors, and changes in placental, endothelial, and kidney biomarkers.^57,58^ The weathering hypothesis states that chronic exposure to social and economic disadvantage leads to an accelerated decline in physical health outcomes and can explain racial disparities in a variety of health conditions.^59^ Most of the literature has found worse ART outcomes in Black women compared with White women; more aggressive ovarian stimulation is required in Black women, and they have longer duration of infertility, higher body mass index, lower implantation rates, higher rates of fibroids, and higher rates of spontaneous abortion.^60^ Black race is a risk factor for placental abruption, and both Black race and placental abruption are risk factors for preterm delivery; yet, among preterm births, the risk of placental abruption is lower in Black women compared with White women after adjusting for socioeconomic status and medical risk factors.^54^ This seemingly contradictory finding suggests that other mechanisms of preterm birth may play a greater role in Black women, such as spontaneous preterm labor or premature rupture of membranes. Most patients undergoing infertility treatment are White or Asian, suggesting a racial disparity between the composition of the population receiving fertility treatment compared with the general population.^61^ There is a lack of data and consistent findings on ART outcomes in the Hispanic population^62^; some studies suggest that Hispanic women with ART conception have similar rates of pregnancy, spontaneous abortion, and live births compared with White women with ART conception.^62^ Hispanic women are less likely to seek or receive infertility treatment and are more likely to have preterm delivery and low-birth-weight infants after ART compared with non-Hispanic White women undergoing ART.^63^

### Limitations

With information from an administrative database using *ICD-9-CM* and *ICD-10-CM* data, we were unable to determine the certainty of a diagnosis and in which hospitalization period it occurred. We included diagnoses of fertility testing, male factor infertility, procreative management, and female infertility but acknowledge that these *ICD-9-CM* and *ICD-10-CM* codes do not fully reflect the use of ART. Procreative management can be used as a diagnosis code for appointments that are not related to infertility. Patients with ART conception were diagnosed primarily with obstetric codes for pregnancy resulting from ART, supervision of pregnancy with a history of infertility, and complications associated with artificial fertilization. The Centers for Disease Control and Prevention estimates that 2.3% of infants born in the US are conceived using ART,^64^ but our study identified an overall prevalence of only 0.5%. This suggests there could be data that are unaccounted for that could have some unknown impact on our results. Our dataset did not specify which specific ART processes were used or the subcategories of each type of ART.

The NIS does not track women across years, so we were unable to account for repeated pregnancy outcomes. Owing to data limitations, we were unable to distinguish between spontaneous and clinician-initiated preterm delivery. Although we found a greater risk of preterm delivery in patients who both conceived via ART and developed placental abruption, it is unclear whether preterm delivery and placental abruption are due to the processes of ART itself or due to diseases related to infertility.

## Conclusions

To our knowledge, this cross-sectional study is the first to elucidate an additive association between the combination of placental abruption and ART conception on subsequent preterm delivery, but there are still unanswered questions about the mechanisms underlying this association. Our findings underscore the importance of counseling patients undergoing the demanding process of infertility treatment on risks to their pregnancy. The adverse effects of infertility treatments are continually being researched to direct potential targeted therapies. Finally, health care practitioners need to be aware of the complex interplay of race and ethnicity on preterm delivery and neonatal outcomes among pregnancies conceived by ART.

## Notes

Supplement 1. **eFigure.** Prevalence of ART Deliveries and the Prevalence of Placental Abruption Among Women who Conceived by ART or Spontaneously: National Inpatient Sample, 2000-2019 **eTable 1.** International Classification of Diseases Codes Used in the Study **eTable 2.** Risks of Placental Abruption in Pregnancies Conceived by ART or Spontaneously, National Inpatient Sample, 2000 to 2019 **eTable 3.** Rates of Preterm Delivery (<37 Weeks’ Gestation) in Pregnancies Conceived by ART or Spontaneously, National Inpatient Sample, 2000 to 2019 **eTable 4.** Rates of Preterm Delivery Among Pregnancies Conceived by ART or Spontaneously, National Inpatient Sample, 2000 to 2019 **eTable 5.** Relationship Between Conception by ART and Placental Abruption, Alone and in Combination, on the Risk of Preterm Delivery, National Inpatient Sample, 2000 to 2019

Supplement 2.
Data Sharing Statement
