# Supplementary material for: Risks of Placental Abruption and Preterm Delivery in Patients Undergoing Assisted Reproduction
Source: JAMA Netw Open. 2024 Jul 10;7(7):e2420970. doi: 10.1001/jamanetworkopen.2024.20970 (PMC11238021; doi:10.1001/jamanetworkopen.2024.20970)
Supplement: Supplement 1. — eFigure. Prevalence of ART Deliveries and the Prevalence of Placental Abruption Among Women who Conceived by ART or Spontaneously: National Inpatient Sample, 2000-2019 eTable 1. International Classification of Diseases Codes Used in the Study eTable 2. Risks of Placental Abruption in Pregnancies Conceived by ART or Spontaneously, National Inpatient Sample, 2000 to 2019 eTable 3. Rates of Preterm Delivery (<37 Weeks’ Gestation) in Pregnancies Conceived by ART or Spontaneously, National Inpatient Sample, 2000 to 2019 eTable 4. Rates of Preterm Delivery Among Pregnancies Conceived by ART or Spontaneously, National Inpatient Sample, 2000 to 2019 eTable 5. Relationship Between Conception by ART and Placental Abruption, Alone and in Combination, on the Risk of Preterm Delivery, National Inpatient Sample, 2000 to 2019 [file jamanetwopen-e2420970-s001.pdf]

# Supplemental Online Content

Zhang J, Lee R, Sauer MV, Ananth CV. Risks of placental abruption and preterm delivery in patients undergoing assisted reproduction. *JAMA Netw Open*. 2024;7(7):e2420970. doi:10.1001/jamanetworkopen.2024.20970

**eFigure 1.** Prevalence of ART Deliveries and the Prevalence of Placental Abruption Among Women who Conceived by ART or Spontaneously: National Inpatient Sample, 2000-2019

**eTable 1.** *International Classification of Diseases* Codes Used in the Study

**eTable 2.** Risks of Placental Abruption in Pregnancies Conceived by ART or Spontaneously, National Inpatient Sample, 2000 to 2019

**eTable 3.** Rates of Preterm Delivery (<37 Weeks' Gestation) in Pregnancies Conceived by ART or Spontaneously, National Inpatient Sample, 2000 to 2019

**eTable 4.** Rates of Preterm Delivery Among Pregnancies Conceived by ART or Spontaneously, National Inpatient Sample, 2000 to 2019

**eTable 5.** Relationship Between Conception by ART and Placental Abruption, Alone and in Combination, on the Risk of Preterm Delivery, National Inpatient Sample, 2000 to 2019

This supplemental material has been provided by the authors to give readers additional information about their work.

**eFigure 1**
**Overall Prevalence of ART Deliveries and Prevalence of Placental Abruption Among Patients Based on Assisted Reproduction Technology Over Time, National Inpatient Sample, 2000 to 2019**

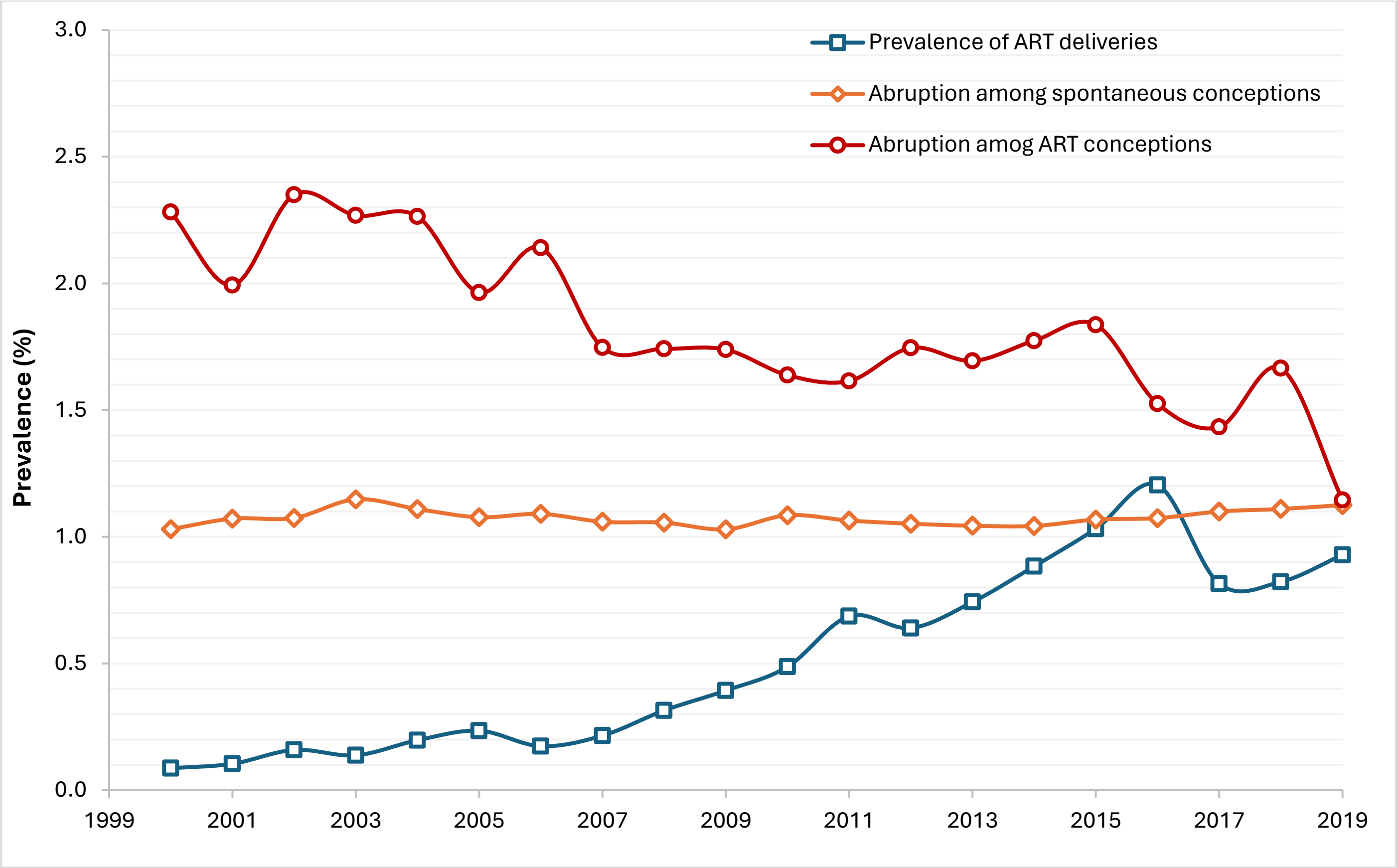


© 2024 Zhang J et al. *JAMA Network Open.*

**eTable 1**
***International Classification of Diseases*** **Codes Used in the Study**

| Description | ICD-9 | ICD-10 | Number of cases (rate) |
|---|---|---|---|
| **Inclusion Criteria** | | | **Number cases (rate) per 100 delivery hospitalizations** |
| Delivery | V27, 650, 720[a], 721[a], 7221[a], 7229[a], 7231[a], 7239[a], 724[a], 726[a], 7251[a], 7252[a], 7253[a], 7254[a], 7271[a], 7279[a], 728[a], 729[a], 7322[a] | Z37, O80, 10D07Z3[a], 0W8NXZZ[a], 10D07Z4[a], 10D07Z5[a], 10S07ZZ[a], 10D07Z3[a], 10D07Z4[a], 10D07Z5[a], 10D07Z6[a], 0W8NXZZ[a], 10D07Z6[a], 10D07Z8[a], 10D07Z7[a], | 78,901,058 (100) |
| Cesarean Section | 649.8, 669.70, 669.71, 740[a], 741[a], 742[a], 744[a], 7499[a] | O82, O75.82 10D00Z0[a], 10D00Z1[a], 10D00Z2[a] | 24,579,354 (31) |
| Multiple Births | V27.2-V27.7, 651 | Z37.2-Z37.7, O30 | 1,410,106 (2) |
| **Exclusion Criteria** | | | |
| Ectopic and Molar Pregnancy and other pregnancies with abortive outcomes | 630-639 6901[a], 6951[a], 7491[a], 750[a] | O00, O01, O02, O03, O04, O07, O08, Z33.2 10A07ZZ[a], 10A08ZZ[a], 10A00ZZ[a], 10A03ZZ[a], 10A04ZZ[a], 10A07ZX[a] | - |

| Description | ICD-9 | ICD-10 | # of cases (rate) |
|---|---|---|---|
| **Exposure** | | | |
| **Infertility Treatment Diagnosis Codes** | | | **# cases (rate) per 100,000 delivery hospitalizations** |

© 2024 Zhang J et al. *JAMA Network Open.*

| | | | |
|---|---|---|---|
| **Encounter for other procreative management** | Z31.8 | | **Total encounter visits** |
| Encounter for male factor infertility in female patient | Z31.81 | V26.89 | 10,202 (13) |
| **Encounter for gamete intrafallopian transfer** | Z31.83 | V26.81 | |
| **Encounter for assisted reproductive fertility procedure cycle** | Z31.83 | V26.81 | |
| Encounter for fertility preservation procedure | | | |
| **Encounter for other procreative management (intrauterine** | Z31.84 | V26.82 | |
| **insemination cycle, artificial insemination)** | Z31.89 | V26.1/V26.89 | |
| Encounter for procreative management and counseling for gestational carrier | Z31.7 | V26.89 | |
| Encounter for procreative management, unspecified | Z31.9 | V26.9 | |
| **Procreation management investigation and testing** | Z31.4 | V26.2 | **Total Procreation visits** |
| Fertility testing | Z31.41 | V26.21 | 286 (0.4) |
| Aftercare following sterilization reversal | Z31.42 | V26.22 | |
| Encounter for other procreative investigation/testing | Z31.49 | V26.29 | |
| Encounter for general counseling and procreation advice | Z31.6 | | |
| Procreative counseling/advice using natural family planning | Z31.61 | V26.41 | |
| Encounter for fertility preservation counseling | Z31.62 | V26.42 | |
| Encounter for other general counseling and advice on procreation | Z31.69 | V26.49 | |
| **Pregnancy resulting from assisted reproductive technology** | O09.81 | V23.85 | **Total pregnancy resulting from ART** |
| Unspecified trimester | O09.819 | V23.85 | 107,326 (136) |
| In first trimester | O09.811 | V23.85 | |
| In second trimester | O09.812 | V23.85 | |
| In third trimester | O09.813 | V23.85 | |

| **Description** | **ICD-9** | **ICD-10** | **# of cases (rate)** |
|---|---|---|---|
| **Supervision of pregnancy with history of infertility** | O09.0 | V23.0 | **Total supervision of pregnancy** |
| Unspecified trimester | O09.00 | V23.0 | 88,552 (112) |
| First trimester | O09.01 | V23.0 | |
| Second trimester | O09.02 | V23.0 | |
| Third trimester | O09.03 | V23.0 | |
| **Complications associated with artificial fertilization** | N98 | - | **Total complications associated with ART** |
| Infection associated with artificial insemination | N98.0 | - | |

© 2024 Zhang J et al. *JAMA Network Open.*

| | | | |
|---|---|---|---|
| Hyperstimulation of ovaries | N98.1 | 256.1 | 203,351 (258) |
| Complications of attempted in vitro fertilization | N98.2 | - | |
| Complications of embryo transfer | N98.3 | - | |
| Other complications associated with artificial fertilization | N98.8 | - | |
| Disorder due-artificial insemination | N98.9 | - | |
| | | | |
| Exam of potential donor of organ or tissue | Z00.5 | V70.8 | **Total Potential donor** 18 (0) |
| | | | |
| Egg Donor | | | **Total Egg Donor** |
| Egg Donor, unspecified | Z52.819 | V59.70 | 102 (0.1) |
| Egg Donor, < 35, anonymous | Z52.810 | V59.71 | |
| Egg Donor, <35, designated | Z52.811 | V59.72 | |
| Egg Donor, 35+, anonymous | Z52.812 | V59.73 | |
| Egg Donor, 35+, designated | Z52.813 | V59.74 | |
| Female Infertility associated with anovulation | N97.0 | 628.0 | |
| Female Infertility of hypothalamic pituitary origin | E23.0 | 628.1 | |
| Female Infertility of tubal origin | N97.1 | 628.2 | |
| Female Infertility of uterine origin | N97.2 | 628.3 | |
| Female Infertility of cervical/vaginal origin | N97.8 | 628.4 | |
| Female Infertility of other origin | N97.8 | 628.8 | |
| Female Infertility, unspecified | N97.9 | 628.9 | |
| Female Infertility associated with Stein-Leventhal Syndrome | E28.2 | 256.4 | |
| Tuboplasty, post previous sterilization | Z31.0 | V26.0 | **Total Tuboplasty** 746 (1) |

| **Description** | **ICD-9** | **ICD-10** | **# of cases (rate)** |
|---|---|---|---|
| **Outcome** | | | **# cases (rate) per 100 delivery hospitalizations** |
| Placental abruption | 641.2 | O45 | 850,680 (1) |
| Preterm delivery | 644.21, 765.2 | O60, Z3A.20-Z3A.36 | 6,106,757 (8) |

[a] Procedure Codes. Delivery-related procedures for forceps, breech extraction, vacuum extraction, other specified and unspecified delivery, and internal and combined version and extraction. Exclusion criteria procedures for pregnancy with abortive outcomes.

ART, Assisted Reproductive Technology
[a] Procedure Codes. Delivery-related procedures for forceps, breech extraction, vacuum extraction, other specified and unspecified delivery, and internal and combined version and extraction. Exclusion criteria procedures for pregnancy with abortive outcomes.

© 2024 Zhang J et al. *JAMA Network Open.*

**eTable 2**
**Risks of Placental Abruption in Pregnancies Conceived by ART or Spontaneously, National Inpatient Sample, 2000 to 2019**

| Characteristic | Assisted reproduction | | Spontaneous conceptions | | Risk difference (95% confidence interval) per 1000[a] | Odds ratio (95% confidence interval) | |
|---|---|---|---|---|---|---|---|
| | Total delivery hospitalizations | Abruption (Risk per 1000) | Total delivery hospitalizations | Abruption (Risk per 1000) | | Unadjusted | Adjusted |
| Singleton[b] | 336,512 | 5,053 (15) | 77,154,440 | 813,719 (11) | 5 (4 to 6) | 1.43 (1.34 to 1.53) | 1.46 (1.37 to 1.56) |
| Multiple[b] | 55,268 | 1,535 (28) | 1,354,838 | 30,373 (22) | 5 (2 to 9) | 1.25 (1.10 to 1.41) | 1.28 (1.13 to 1.45) |
| Age 15-19[c] | 2,013 | -[e] | 6,749,449 | 72,971 (11) | - | - | - |
| Age 20-24[c] | 22,910 | 304 (13) | 18,258,807 | 190,710 (10) | 3 (0 to 6) | 1.28 (0.99 to 1.64) | 1.27 (0.99 to 1.63) |
| Age 25-29[c] | 84,224 | 1,403 (17) | 21,916,044 | 224,436 (10) | 7 (5 to 9) | 1.66 (1.47 to 1.87) | 1.63 (1.45 to 1.84) |
| Age 30-34[c] | 141,113 | 2,357 (17) | 19,697,130 | 208,615 (11) | 6 (5 to 8) | 1.59 (1.44 to 1.74) | 1.56 (1.42 to 1.72) |
| Age 35-39[c] | 98,579 | 1,649 (17) | 9,697,907 | 116,610 (12) | 5 (3 to 7) | 1.40 (1.25 to 1.57) | 1.36 (1.21 to 1.53) |
| Age 40-44[c] | 34,297 | 681 (20) | 2,067,052 | 31,584 (15) | 5 (1 to 8) | 1.31 (1.10 to 1.56) | 1.32 (1.10 to 1.57) |
| Age 45-54[c] | 8,644 | 188 (22) | 122,890 | 2,166 (18) | 4 (-3 to 11) | 1.24 (0.88 to 1.76) | 1.18 (0.82 to 1.68) |
| Low[d] | 51,207 | 847 (17) | 18,811,942 | 229,692 (12) | 4 (2 to 7) | 1.36 (1.17 to 1.59) | 1.27 (1.09 to 1.48) |
| Medium-low[d] | 73,684 | 1,073 (15) | 19,024,702 | 209,892 (11) | 4 (2 to 6) | 1.33 (1.1 to 1.52) | 1.20 (1.04 to 1.37) |
| Medium-high[d] | 103,790 | 1,680 (16) | 19,122,630 | 198,733 (10) | 6 (4 to 8) | 1.57 (1.40 to 1.75) | 1.37 (1.22 to 1.54) |
| High[d] | 159,723 | 2,925 (18) | 20,332,096 | 191,889 (9) | 9 (7 to 11) | 1.96 (1.79 to 2.15) | 1.62 (1.48 to 1.78) |

[a]Risks are expressed per 1000 delivery hospitalizations.
[b]Odds ratios by multiple gestation were adjusted for year, age group, hospital bed size, hospital teaching status, location, region, mother's race/ethnicity, insurance, and income based on a survey logistic regression model.
[c]Odds ratios by age group were adjusted for year, hospital bed size, hospital teaching status, location, region, mother's race/ethnicity, multiple birth, insurance, and income based on a survey logistic regression model.
[d]Odds ratios by income were adjusted for year, age group, hospital bed size, hospital teaching status, location, region, mother's race/ethnicity, multiple birth, and insurance based on a survey logistic regression model.
[e]Number suppressed since cell count < 10-comply with HCUP data use agreement

© 2024 Zhang J et al. *JAMA Network Open.*

**eTable 3**
**Rates of Preterm Delivery (<37 Weeks' Gestation) in Pregnancies Conceived by ART or Spontaneously, National Inpatient Sample, 2000 to 2019**

| Characteristic | Assisted reproduction | | Spontaneous conception | | Risk difference (95% confidence interval) | Odds ratio (95% confidence interval) | |
|---|---|---|---|---|---|---|---|
| | Total delivery hospitalizations | Preterm delivery (Risk per 1000) | Total delivery hospitalizations | Preterm delivery (Risk per 1000) | | Unadjusted | Adjusted |
| Singleton[a] | 336,512 | 36,385 (108) | 77,154,440 | 5,385,789 (70) | 38 (36 to 41) | 1.62 (1.57 to 1.67) | 1.62 (1.57 to 1.67) |
| Multiple[a] | 55,268 | 27,340 (495) | 1,354,838 | 657,224 (485) | 10 (-2 to 22) | 1.04 (0.99 to 1.09) | 1.21 (1.15 to 1.27) |
| Age 15-19[b] | 2,013 | 215 (107) | 6,749,449 | 582,900 (86) | 20 (-11 to 51) | 1.26 (0.94 to 1.75) | 1.12 (0.81 to 1.56) |
| Age 20-24[b] | 22,910 | 3,340 (146) | 18,258,807 | 1,409,639 (77) | 69 (58 to 79) | 2.04 (1.88 to 2.22) | 1.65 (1.51 to 1.79) |
| Age 25-29[b] | 84,224 | 12,923 (153) | 21,916,044 | 1,576,561 (72) | 82 (75 to 88) | 2.34 (2.23 to 2.45) | 1.62 (1.55 to 1.70) |
| Age 30-34[b] | 141,113 | 22,707 (161) | 19,697,130 | 1,446,532 (73) | 88 (81 to 94) | 2.42 (2.31 to 2.53) | 1.59 (1.52 to 1.66) |
| Age 35-39[b] | 98,579 | 16,695 (169) | 9,697,907 | 805,342 (83) | 86 (80 to 93) | 2.25 (2.15 to 2.36) | 1.46 (1.39 to 1.53) |
| Age 40-44[b] | 34,297 | 6,062 (177) | 2,067,052 | 204,318 (99) | 78 (68 to 88) | 1.96 (1.83 to 2.10) | 1.35 (1.26 to 1.46) |
| Age 45-54[b] | 8,644 | 1,782 (206) | 122,890 | 17,741 (144) | 62 (40 to 84) | 1.54 (1.34 to 1.76) | 1.13 (0.96 to 1.32) |
| Low[c] | 51,207 | 8,477 (166) | 18,811,942 | 1,689,942 (90) | 76 (67 to 84) | 2.01 (1.89 to 2.14) | 1.45 (1.36 to 1.54) |
| Medium-low[c] | 73,684 | 11,638 (158) | 19,024,702 | 1,476,122 (78) | 80 (73 to 87) | 2.23 (2.11 to 2.35) | 1.48 (1.41 to 1.56) |
| Medium-high[c] | 103,790 | 16,858 (162) | 19,122,630 | 1,402,639 (73) | 89 (83 to 96) | 2.45 (2.34 to 2.57) | 1.49 (1.42 to 1.56) |
| High[c] | 159,723 | 26,066 (163) | 20,332,096 | 1,376,043 (68) | 96 (89 to 102) | 2.69 (2.57 to 2.82) | 1.43 (1.38 to 1.50) |

[a]Odds ratios by multiple gestation were adjusted for year, age group, hospital bed size, hospital teaching status, location, region, mother's race/ethnicity, insurance, and income based on a survey logistic regression model.
[b]Odds ratios by age group were adjusted for year, hospital bed size, hospital teaching status, location, region, mother's race/ethnicity, multiple birth, insurance, and income based on a survey logistic regression model.
[c]Odds ratios by income were adjusted for year, age group, hospital bed size, hospital teaching status, location, region, mother's race/ethnicity, multiple birth, and insurance based on a survey logistic regression model

© 2024 Zhang J et al. *JAMA Network Open.*

**eTable 4**

**Rates of Preterm Delivery Among Pregnancies Conceived by ART or Spontaneously, National Inpatient Sample, 2000 to 2019**

| Characteristic | Assisted reproduction only | | Spontaneous conception | | Placental abruption only | | Both assisted reproduction and placental abruption | |
|---|---|---|---|---|---|---|---|---|
| | Total delivery hospitalizations | Preterm delivery (Risk per 1000) | Total delivery hospitalizations | Preterm delivery (Risk per 1000) | Total pregnancies | Preterm delivery (Risk per 1000) | Total delivery hospitalizations | Preterm delivery (Risk per 1000) |
| Singleton | 331,459 | 33,906 (102) | 76,340,721 | 5,052,391 (66) | 813,719 | 333,397 (410) | 5,053 | 2,479 (491) |
| Multiple | 53,732 | 26,159 (487) | 1,324,464 | 635,235 (480) | 30,373 | 22,009 (725) | 1,535 | 1,180 (769) |
| Age 15-19 | 2,008 | 210 (104) | 6,676,479 | 547,602 (82) | 72,971 | 35,298 (484) | -[a] | - |
| Age 20-24 | 22,605 | 3,156 (140) | 18,068,097 | 1,325,295 (73) | 190,710 | 84,344 (442) | 304 | 185 (607) |
| Age 25-29 | 82,821 | 12,156 (147) | 21,694,607 | 1,484,733 (68) | 221,436 | 91,829 (415) | 1,403 | 768 (547) |
| Age 30-34 | 138,756 | 21,366 (154) | 19,488,515 | 1,361,978 (70) | 208,615 | 84,554 (405) | 2,357 | 1,341 (569) |
| Age 35-39 | 96,929 | 15,815 (163) | 9,581,297 | 759,143 (79) | 116,610 | 46,199 (396) | 1,649 | 881 (534) |
| Age 40-44 | 33,616 | 5,698 (169) | 2,035,467 | 192,042 (94) | 31,584 | 12,275 (389) | 681 | 364 (534) |
| Age 45-54 | 8,455 | 1,666 (197) | 120,724 | 16,833 (139) | 2,166 | 908 (419) | 188 | 117 (618) |
| Low | 50,360 | 8,005 (159) | 18,582,249 | 1,585,160 (85) | 229,692 | 104,782 (456) | 847 | 472 (557) |
| Medium-low | 72,611 | 11,004 (151) | 18,814,810 | 1,387,371 (74) | 209,892 | 88,751 (423) | 1,073 | 634 (590) |
| Medium-high | 102,110 | 15,943 (156) | 18,923,897 | 1,322,128 (70) | 198,733 | 80,510 (405) | 1,680 | 914 (544) |
| High | 156,798 | 24,462 (156) | 20,140,207 | 1,300,397 (65) | 191,889 | 75,646 (394) | 2,925 | 1,604 (548) |

[a]Number suppressed since cell count <10 to comply with the HCUP data use agreement

© 2024 Zhang J et al. *JAMA Network Open.*

**eTable 5**
**Relationship Between Conception by ART and Placental Abruption, Alone and in Combination, on the Risk of Preterm Delivery, National Inpatient Sample, 2000 to 2019**

| Characteristic | Assisted reproduction only | | Placental abruption only | | Both assisted reproduction and abruption | | RERI[d] (95% CI) |
|---|---|---|---|---|---|---|---|
| | Unadjusted odds ratio (95% CI) | Adjusted odds ratio (95% CI) | Unadjusted odds ratio (95% CI) | Adjusted odds ratio (95% CI) | Unadjusted odds ratio (95% CI) | Adjusted odds ratio (95% CI) | |
| Singleton[a] | 1.61 (1.56 to 1.66) | 1.61 (1.56 to 1.65) | 9.79 (9.64 to 9.95) | 9.52 (9.37 to 9.57) | 13.59 (11.97 to 15.4) | 13.88 (12.20 to 15.80) | 3.8 (2.0 to 5.5) |
| Multiple[a] | 1.03 (0.98 to 1.08) | 1.20 (1.14 to 1.26) | 2.86 (2.70 to 3.02) | 2.87 (2.71 to 3.04) | 3.60 (2.76 to 4.70) | 4.26 (3.27-5.54) | 1.2 (0.1 to 2.3) |
| Age 15-19[b] | 1.31 (0.94 to 1.81) | 1.16 (0.83 to 1.61) | 10.49 (10.11 to 10.87) | 10.47 (10.09 to 10.87) | - | - | - |
| Age 20-24[b] | 2.05 (1.88 to 2.23) | 1.65 (1.51 to 1.80) | 10.02 (9.77 to 10.27) | 10.04 (9.79 to 10.29) | 19.44 (11.63 to 32.47) | 15.98 (9.48 to 26.96) | 5.3 (-3.1 to 13.6) |
| Age 25-29[b] | 2.34 (2.23 to 2.46) | 1.61 (1.53 to 1.69) | 9.64 (9.43 to 9.87) | 9.73 (9.50 to 9.96) | 16.44 (12.97 to 20.83) | 12.94 (9.80 to 17.09) | 2.6 (-1.0 to 6.2) |
| Age 30-34[b] | 2.42 (2.32 to 2.54) | 1.57 (1.50 to 1.64) | 9.07 (8.86 to 9.28) | 9.17 (8.95 to 9.39) | 17.54 (14.55 to 21.15) | 13.43 (10.92 to 16.51) | 3.7 (0.9 to 6.5) |
| Age 35-39 [b] | 2.27 (2.16 to 2.38) | 1.45 (1.38 to 1.52) | 7.63 (7.41 to 7.85) | 7.71 (7.47 to 7.95) | 13.31 (10.71 to 16.55) | 10.02 (7.78 to 12.89) | 1.9 (-0.7 to 4.4) |
| Age 40-44 [b] | 1.96 (1.83 to 2.10) | 1.34 (1.24 to 1.44) | 6.10 (5.78 to 6.45) | 6.26 (5.90 to 6.63) | 11.02 (8.02 to 15.14) | 9.73 (6.57 to 14.39) | 3.1 (-0.7 to 7.0) |
| Age 45-54 [b] | 1.51 (1.33 to 1.73) | 1.10 (0.94 to 1.29) | 4.45 (3.65 to 5.42) | 4.70 (3.77 to 5.85) | 9.99 (4.98 to 20.04) | 9.14 (4.17 to 20.05) | 4.3 (-2.9 to 11.5) |
| Low[c] | 2.03 (1.90 to 2.16) | 1.46 (1.37 to 1.55) | 9.00 (8.78 to 9.22) | 9.04 (8.82 to 9.27) | 13.50 (0.86 to 18.50) | 9.67 (6.64- to 14.10) | 0.2 (-3.5 to 3.8) |
| Medium-low[c] | 2.24 (2.13 to 2.37) | 1.48 (1.40 to 1.56) | 9.20 (8.98 to 9.43) | 9.28 (0.05 to 9.51) | 18.08 (13.57 to 24.11) | 14.81 (10.88 to 20.17) | 5.1 (0.5 to 9.6) |
| Medium-high[c] | 2..46 (2.35 to 2.58) | 1.48 (1.41 to 1.55) | 9.07 (8.85 to 9.29) | 9.21 (8.99 to 9.43) | 15.90 (12.87 to 19.64) | 11.18 (8.74 to 14.31) | 1.5 (-1.3 to 4.2) |
| High[c] | 2.68 (2.56 to 2.80) | 1.41 (1.36 to 1.47) | 9.43 (9.19 to 9.67) | 9.67 (9.41 to 9.93) | 17.58 (14.73 to 20.99) | 12.00 (9.80 to 14.69) | 1.9 (-0.4 to 4.3) |

Abbreviations: CI, confidence interval; RERI, relative excess risk due to interaction

[a]Odds ratios by multiple gestations were adjusted for year, age group, hospital bed size, hospital teaching status, location, region, mother's race/ethnicity, insurance, and income based on a survey logistic regression model.
[b]Odds ratios by age group were adjusted for year, hospital bed size, hospital teaching status, location, region, mother's race/ethnicity, multiple birth, insurance, and income based on a survey logistic regression model.
[c]Odds ratios by income were adjusted for year, age group, hospital bed size, hospital teaching status, location, region, mother's race/ethnicity, multiple birth, and insurance based on a survey logistic regression model.
[d]RERI = $RR_{++} - RR_{+-} - RR_{-+} + 1$

© 2024 Zhang J et al. *JAMA Network Open.*
