# Supplementary material for: Risks of Placental Abruption and Preterm Delivery in Patients Undergoing Assisted Reproduction
Source: JAMA Netw Open. 2024 Jul 10;7(7):e2420970. doi: 10.1001/jamanetworkopen.2024.20970 (PMC11238021; doi:10.1001/jamanetworkopen.2024.20970)
Supplement: Supplement 2. — Data Sharing Statement [file jamanetwopen-e2420970-s002.pdf]

Data Sharing Statement

Zhang. Risks of Placental Abruption and Preterm Delivery in Patients Undergoing Assisted Reproduction. *JAMA Netw Open*. Published July 10, 2024.
doi:10.1001/jamanetworkopen.2024.20970

Data
Data available: No

Additional Information
Explanation for why data not available: All data utilised in this study can be accessed from the Healthcare Cost and Utilization Project (https://www.hcup-us.ahrq.gov/).
